# Medical Items

**Published:** 1907-10

**Authors:** 


### MEDICAL ITEMS.

Mr. Andrew Carnegie has unconditionally contributed $500.000 to King Edward's Hospital fund.

---

Bouchard has truly said "What makes possible the development of an infectious disease is not the chance meeting of microbe and man." This is occurring constantly, but there must be, in addition, a condition of the body favorable to the development and multiplication of the invading germ.

One of the most common conditions which so lowers the resisting powers of the system as to permit bacterial invasion is the toxaemia of faulty metabolism. The ideal treatment of toxaemia is by elimination. Alkalithia is the ideal eliminant and its use in selected cases of rheumatism, asthma, tonsillitis, cholera, eczema, urticaria, pruritus and incipient Bright's Disease will well repay a careful trial.

---

The seventh annual meeting of the board of governors of the Georgia Pasteur Institute was held in the office of the institute, at 85 Luckie St., Sept. 19th.

The report of Dr. Jas. N. Brawner, physician in charge, showed that up to date 650 patients have been treated, of whom only two have died, after the completion of the time of treatment.

The following officers were elected:

Henry R. Slack, M. D., President, LaGrange, Ga.

J. H. McDuffie, M. D., Vice-President, Columbus, Ga.

Benjamin W. Hunt, 2nd Vice-President, Eatonton, Ga.

Claud A. Smith, M. D., Pathologist.

Jas. N. Brawner, M. D., Physician in charge.

E. C. Cartledge, M. D., Assistant Physician, Atlanta, Ga.

### THE GREATEST OF ANESTHETICS.

We are living in an age of advancement in medicine as well as in all departments of life, and he who does not hustle today will wake up tomorrow looking into space and wondering where his neighbors are who were with him yesterday.

I have been very much interested in Dr. Abbot's recently discovered hypnotic anasthetic, i: e:. hyoscine, morphine and cactin compound. I have used it in a number of operative cases, with very satisfactory results. The anasthesia was perfect, except in two cases where, in addition, I used a very small amount of chloroform, which completed the anesthesia.

I had none of the postoperative nausea which we so much dread following other anesthetics. In one case of weak heart, where I could not use chloroform, the heart's action seemed to improve while under the influencs of this hyoscine, morphine, and cactin anesthetic.

For about two years prior to using the H-M-C anesthetic I had been using hyoscine and morphine as a hypnotic and analgesic, with such excellent results that I felt that I was not properly equipped without hyoscine and morphine tablets in my hypodermatic case, but had never thought of its becoming an anasthetic.

I feel that the medical fraternity is under great obligations to Dr. Abbot for the discovery and careful preparation of this much-needed and greatest of anesthetics, that is proving such a boon to suffering humanity.

Jacksonville, Ill.

C. C. COCHRAN,

### GASTRO-INTESTINAL AILMENTS OF YOUNG CHILDREN,

BY

B. BROWN, M.D., WAUKEGAN, LIL.

As the hot weather approaches the usual number of gastro-intestinal ailments will confront us and if we be not alert the same mortality of old will occur among our little patients of one and two years. The keynote to success in the management of these cases is to see that correct feeding is enforced and to keep the alimentary canal as clean and nearly aseptic as possible. If this be done much suffering can be obviated and many little lives saved.

Every medical man these days is capable of giving correct advice on infant feeding, the care of bottles, accessories, etc., if he will only take the time and trouble to make the mother understand how important it all is. The doctor's suggestions on this matter are too often regarded as simply platitudes and are not thought of seriously until the child is in the throes of a severe illness. The following clinical reports are illustrations of my usual method of handling the more heated season.

Ethel G., aged ten months, suffered from cholera infantum; bottle fed. Was passing watery stools every few minutes. Temperature had been considerably elevated, but was now slightly abnormal. Mouth and tongue parched. Considerable emaciation and scaphoid abdomen. Circulation weak and respirations labored. In fact, an extreme prostrate condition. Treatment: I put four ounces of Glyco-Thymoline with eight ounces of water and gave it as a high enema, causing it to be retained as long as possible. This was repeated every hour or so until the bowels were thoroughly cleansed and the stools diminised in number. Gave one-tenth grain of calomel every two hours until the discharges showed the characteristic greenish color. Also gave the following:

Elixir Lactopeptine ʒij

Glyco-Thymoline ʒij

Oil Peppermint gtt. j

M. Sig.—20 drops every hour. After eight hours the child was able to take nourishment and retain it. This consisted of cold pasteurized milk diluted with an equal portion of lime water. Child was given all the cold water and lemonade she wanted. She made a good recovery.

Jennie M., aged fourteen months, suffered from gastro-enteritis with much fermentation. Bowels swollen and tympanitic. Fever of a remittent type due to autotoxemia. Child delicate and poorly nourished; still nursing at mother's breast. Mother herself in poor health and in no condition to nourish her child. Treatment: Put the little one on cow's milk diluted with lime water. Three times a week I gave a high enema of a warm saline solution and Glyco-Thymoline, equal parts. Also gave the above prescription, a teaspoonful every four hours. Child steadily improved under this treatment and in six weeks was in a good state of nutrition and health.

MEDICAL SUMMARY,

A telegram regarding yellow fever in Cuba from Governor Magoon on August 23 showed that there were no new cases, either in the city or in Cienfuegos.

---

The San Francisco Board of Health has decided to thoroughly cleanse and disinfect the City and County Hospital, and to wage a war of extermination on fleas and rats in the institution. The buildings will then be thoroughly cleansed and disinfected.

---

### THE USE OF ADRENALIN DURING ETHER ANESTHESIA.

By Charles S. Venable, M. D., Charlottesville, Va.

From the Virginia Medical Semi-Monthly, February 22, 1907.

Recognizing that my experience in the use of Adrenalin during ether anesthesia is but very limited, covering a course of only eighteen cases, and knowing the many fallacies attendant upon too early conclusions, I feel a great hesitancy in making this report. However, owing to the uniform result that has attended its use, I am prompted to do so now.

I found that 25 per cent, aqueous solution of the standard 1 in 1000 gave the best results, and that by first pouring ether in the towel cone and spraying the Adrenalin solution on it, depending on the ether to vaporize it sufficiently for the inhalation, was the best mode of administration. Three to six minute intervals are sufficient for its use and a total of from one-half to one ounce of this solution is enough for an operation lasting from thirty minutes to an hour. The effects are a more uniform etherization, the pulse becoming steadier, slower and of better character more rapidly than under ether alone; respirations are quiet and regular, the bronchial secretions are practically checked, and the progress of the operation is not interrupted.

These cases were not selected, and among them were old alcoholics; two women over sixty, one of them nearly eight years of age. Three were very long, tedious operations, lasting over two hours, and in none of the series was any stimulation required during the anesthesia.

Recovery from the anesthetic was uniformly good; there was practically no post-operative shock, and no stimulation was needed in any one of the cases; only two patients vomited at all and very little nausea was complained of.

From the foregoing facts I conclude that owing to the contraction of the smaller vessels the bronchial glands secrete less mucus, and there is better aeration in the bronchioles and pulmonary vesicles, less ether is required to produce anesthesia and there is less probability of ether pneumonia following. The Adrenalin, acting generally from absorption, is a powerful stimulant; it meterially lessens shock, lessens the capillary ooze at the field of operation, and is of great benefit to the much weakened patient.

### THE CURE OF A CASE OF OSTEOMALACIA.

In an article on the suprarenal glands and osteomalacia, in the Munich. Med. Wochenschrift, 1907, p. 278, L. M. Bossi, of Genoa, describes the almost marvelous cure of a serious case of osteomalacia by subcutaneous injections of Adrenalin. The patient was a multipara, 38 years of age, who was enceinte in the eigth month and had a well defined osteomalacia. After seven hypodermatic injections of Adrenalin, each of which consisted of 1-2 cg. of Adrenalin of the 1:1000 solution, the patient fully recovered.

BUFFALO LITHIA WATERS have been made the subject of exhaustive clinical study and observation, and they owe their reputation to the positive results obtained in certain maladies, for it has never been claimed that they are a specific for every disease. The accompanying testimonial shows that they are used generally with highly, and, not infrequently with the most remarkable results in Uric Acid Diathesis, or Lithaemic Conditions. Gout, Rheumatism, Renal Calculi, Stone in the Bladder. Inflamation of the Bladder, Albuminuria, Bright's Disease, and Diabetes, as well as in Gastro-Intestinal Disorders of Women, Puerperal Eclampsia, Nausea and Vomiting of Pregnancy, Malaria and its Sequalae, Scarlatina, Infectious Diseases, Dipsomania, Impotency and Sterility, Diseases of the Nervous System, Diseases of the Skin, and before and after surgical operations.

Dr. I. Halsted Boyland, Paris, says "that in cases where lithia, soda and potash are indicated, he has obtained far better results from the Buffalo Lithia Waters than from any of the tablets or other preparations of the lithium salts."

### A SUGGESTION.

The new Glyco-Thymoline Eye Bath, which is constructed from a single piece of aluminum, has been found of exceptional service when used as a vessel to heat hypodermic solutions to the proper temperature. This little hint comes from a physician who has frequently found himself wanting just such a device. The Glyco-Thymoline people will be glad to send you one of these cups if you desire it.

